# Supplementary material for: The causal relationship between the human gut microbiota and pyogenic arthritis: a Mendelian randomization study
Source: Front Cell Infect Microbiol. 2024 Nov 26;14:1452480. doi: 10.3389/fcimb.2024.1452480 (PMC11629706; doi:10.3389/fcimb.2024.1452480)
Supplement: Supplementary file 1 [file DataSheet1.zip › mendelian test/ebi-a-GCST90027595.csv_forest.pdf]

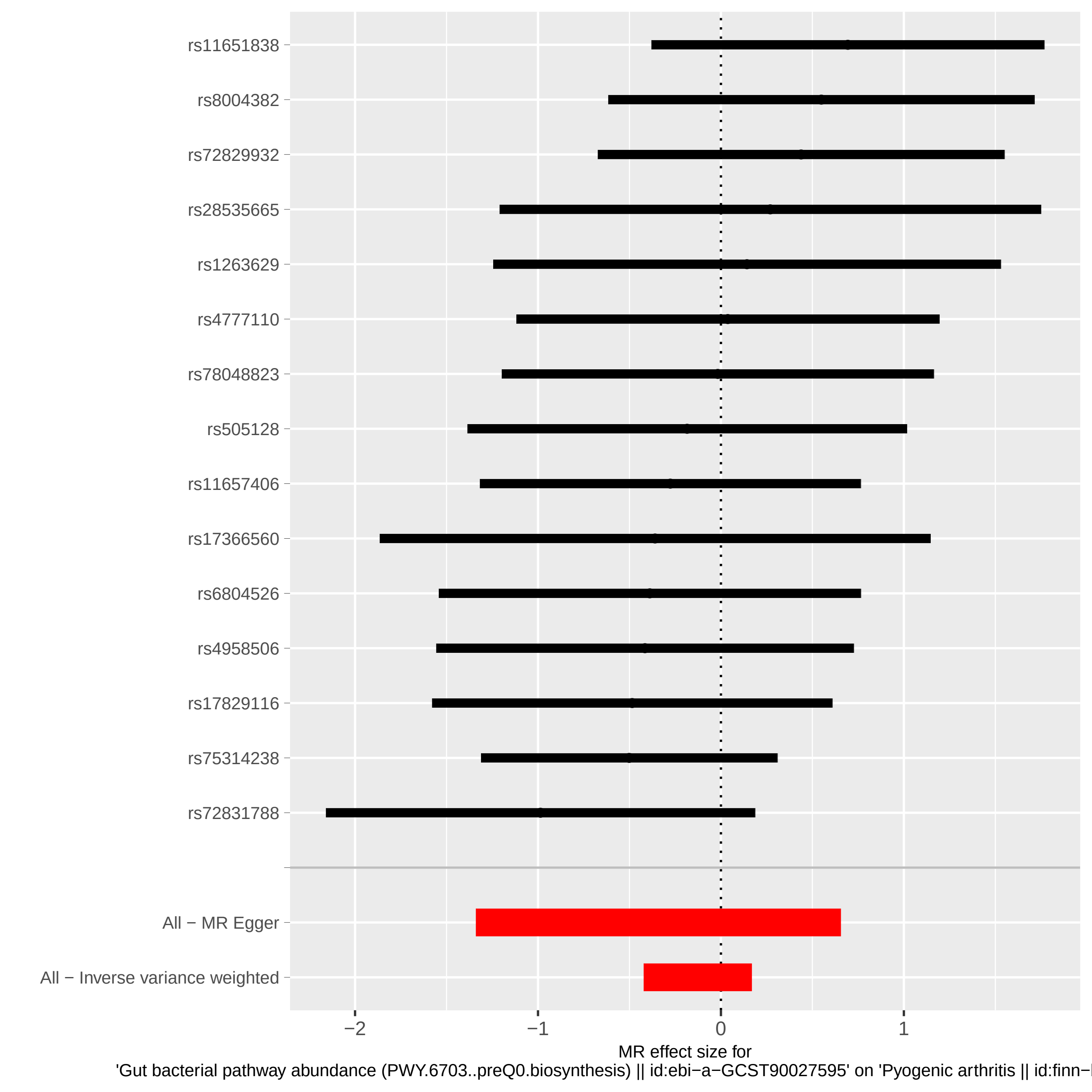
rs11651838
rs8004382
rs72829932
rs28535665
rs1263629
rs4777110
rs78048823
rs505128
rs11657406
rs17366560
rs6804526
rs4958506
rs17829116
rs75314238
rs72831788
All − MR Egger
All − Inverse variance weighted
−2
−1
0
1
MR effect size for
'Gut bacterial pathway abundance (PWY.6703..preQ0.biosynthesis) || id:ebi−a−GCST90027595' on 'Pyogenic arthritis || id:finn−
